# Mitochondrial efficiency impacts nocturnal sheltering in juvenile salmon (*Salmo salar*), affecting the trade-off between foraging and predation risk

**DOI:** 10.1098/rspb.2024.1788

**Published:** 2024-10-30

**Authors:** Neal J. Dawson, Agnieszka Magierecka, Darryl McLennan, Caroline Millet, Jakub Czyzewski, Neil B. Metcalfe

**Affiliations:** ^1^ School of Biodiversity, One Health and Veterinary Medicine, Graham Kerr Building, University of Glasgow, Glasgow G12 8QQ, UK; ^2^ Bioelectronics Unit, College of Medical, Veterinary and Life Sciences, Graham Kerr Building, University of Glasgow, Glasgow G12 8QQ, UK

**Keywords:** optimal foraging theory, ectotherms, ADP/O, global warming, climate change, fish

## Abstract

At cold winter temperatures, juvenile salmonids typically spend much of their time sheltering from predators, which negatively impacts foraging for food. Previous work shows that inter-individual variation in mitochondrial efficiency explains variation in food intake, growth and metabolic rate. Here, we examine whether inter-individual variation in mitochondrial efficiency predicts sheltering as a proxy of foraging patterns for overwintering juvenile Atlantic salmon (*Salmo salar*). PIT-tagged salmon were housed individually under winter conditions, and their use of a custom-built shelter was recorded automatically. In line with the previous research and estimates of relative predation risk, fish showed a broad preference for sheltering during the day and emerging to feed at night. However, they exhibited marked among-individual variation in their use of shelter, which was unrelated to body size but was predicted by mitochondrial function: there was a positive relationship between muscle mitochondrial phosphorylation efficiency and proportion of time spent in the shelter during the night but not during the day. Individuals with the most efficient mitochondria at producing ATP were thus able to spend more time sheltering from predators. This suggests that individual heterogeneity in cellular function may drive variation in the trade-off between foraging and sheltering, which has implications for selection pressures acting on wild populations.

## 1. Introduction

Animals often face a trade-off between the need to acquire resources and the need to minimize their immediate risk of mortality, for instance, through being caught by a predator. This leads to complex behavioural decisions about where and when to feed, based on the relative costs and benefits of different options [1]. One factor that influences these decisions is the energetic or nutritional state of the individual, since options that incur a lower risk of predation may also only provide a lower foraging profitability. A recent meta-analysis showed that animals often demonstrate an ability to match their risk-taking to their nutritional condition [2]. A potential cause of behavioural variation is therefore their rate of energy consumption, since individuals with higher costs of living (the energy required to sustain growth, movement, reproduction, etc.) may need to forage more intensively and so incur greater risks of predation [3,4]. Such behavioural decisions are especially apparent when animals can use a refuge (that provides no access to food) in order to avoid predation: it has therefore been suggested that individuals with a lower energy demand should spend a greater proportion of their time in a refuge [5,6].

The evidence that individual variation in the cost of living is driving behavioural decisions on risk-taking has until now been indirect. The link between cost of living and behaviour comes from measurements of body size, such as mass [5,6], or of whole-animal respiration rates [3,4], which do not take account of the significant variation in mitochondrial efficiency that exists between individual animals of the same species [7]. Indeed, there is increasing evidence that inter-individual variation in mitochondrial function underlies some of the observed variation in the cost of living, including mitochondrial fission and fusion events linked with development and ageing [8] and standard metabolic rate [9], and that this variation strongly correlates with inter-individual variation in growth performance [10,11]. However, it has not been shown whether inter-individual variation in the efficiency with which mitochondria can convert oxygen and substrates into ATP (hereafter ‘mitochondrial efficiency’) explains variation in risk-taking to support the cost of living, which would result in a link between cellular efficiency and behaviour.

Juvenile salmonid fish such as the Atlantic salmon (*Salmo salar*) provide an ideal system to examine this potential relationship between anti-predator behaviour and cellular energy production efficiency. They enter a state of natural anorexia when temperatures drop in winter, hiding from predators in streambed refuges and only emerging to feed enough to keep to a programmed trajectory of fat loss; this reduces their overall feeding time and hence exposure to predators [12]. They are more likely to hide in the refuges during the day than at night, since the predation risk at night is much reduced [13]. However, the proportion of time spent out of the refuge and foraging is greater when the fish have lower nutritional reserves [14] and when the food availability is lower, so that time out of refuge is correlated with foraging requirements [13]. The empirical results fit the predictions of a model in which the fish are optimizing their use of refuges so that they balance the need to feed against the risk of predation [15].

In this study, we conducted the first test (to our knowledge) of whether mitochondrial physiology can explain inter-individual variation in behaviour. We tested whether mitochondrial ATP production efficiency influences the behavioural trade-off between sheltering from predators and the need to emerge to seek food. Owing to the positive relationship between mitochondrial efficiency and growth rates in salmonids [10,11], it can be hypothesized that animals with a higher mitochondrial efficiency will exhibit lower rates of fuel consumption, since less substrate and O_2_ will need to be consumed to produce each molecule of ATP. This should allow them to reduce their foraging times and increase their time spent sheltering, ultimately lowering any predation risk. However, it is known that greater mitochondrial efficiency can come at a cost of a higher rate of production of reactive oxygen species (ROS), which can cause oxidative damage to biomolecules [16,17]. We therefore related mitochondrial respiration, mitochondrial energy production efficiency and ROS release rates in juvenile Atlantic salmon to time spent sheltering under winter conditions. We hypothesized that greater mitochondrial efficiency will reduce the amount of time an individual spends out of a shelter, since it requires less food. We also predicted that fish would spend more time sheltering during the day—when there would typically be a greater risk of predation—than during the night. Lastly, we predicted that higher mitochondrial efficiency may come at a cost of increased ROS release rates from the mitochondria.

## 2. Material and methods

### (a) Animal collection and husbandry

Wild sexually mature adult salmon (*Salmo salar*; initial wet mass 1.41–3.81 kg; fork length 51–70 cm; *n* = 6 males and 6 females) were caught by electrofishing when they were migrating to the spawning grounds in rivers in the Galloway region, south-west Scotland, by Galloway Fisheries Trust staff in November 2019. Eggs were stripped from ripe females and mixed with expressed sperm (one male fertilizing eggs from one female) as part of hatchery operations, and the fertilized eggs from the resulting six full-sib families (*n* = 50 per family) were reared under ambient temperatures (6 ± 1.5°C) at the Galloway Fisheries Trust hatchery until they were transferred to the University of Glasgow on 18 March 2020, where they were held in a recirculation system supplied with dechlorinated tap water at 11°C. All animal husbandry and subsequent experiments were undertaken under UK Home Office project license P89482164 and following local ethical review. Once the fish had reached the first feeding stage, they were reared in full-sib groups (*n* = 6 groups) in compartments of a stream tank on the same water recirculation system at 11°C under a 12 L : 12 D photoperiod. They were fed daily with commercial fry food (ZM-100 fry food; Zebrafish Management Ltd., Twyford, UK) supplemented with sufficient bloodworms (Red mosquito Bloodworm; Small, Oss, Netherlands) to provide 1–2 bloodworms per fish per day. Once established on exogenous food, all of the young fish were placed in a single circular stock tank and reared on commercial salmon feed pellets.

In April 2021, 55 of these juvenile salmon (initial wet mass 3.73–8.50 g; fork length 74.4–92.1 mm) were randomly selected, weighed and PIT tagged, then transferred to individual tanks (dimensions: 32.5 L × 17.5 W × 17.5 D cm) under a winter (10 L : 14 D) photoperiod, with a low-level night light to simulate moonlight. Fish were allowed to acclimate for one week in the same tanks connected to a recirculation system supplied with dechlorinated tap water at 7.86 ± 0.49°C. The water temperature and photoperiod were chosen to be realistic for winter streams in the source catchment of the fish in southern Scotland [18]. Between 10 and 11 am each day, the tanks were each supplied with an ad libitum level of food (1.5 ml of pre-prepared live *Artemia nauplii* (reared for 2 days in a 1000 ml hatcher filled with water, 500 mM NaCl and approx. 2.5 g l^–1^ *Artemia* eggs bubbled vigorously with air; FirstBite Super Hatch, BCUK Aquatics, Louth, UK), supplemented with 8–10 bloodworms). Tanks were cleaned each day before feeding, and there was often leftover, uneaten bloodworm. This food regime was maintained throughout the experiment. Each tank contained a single 3D-printed tubular opaque plastic refuge in which a PIT tag ring antenna was embedded. The antenna was linked to a custom-made radio frequency identification (RFID) reader that recorded at 16 second intervals when the fish was within the refuge. Data were collected continuously over an approximately one-week period (mean = 164.01 ± 0.23 hours), providing a complete record of the proportion of time spent in the refuge at each time of day (see electronic supplementary material, 1, for detailed information about the construction of this bespoke refuge with a built-in RFID reader).

After the one-week refuge monitoring period, mitochondrial function was measured in the myotome white muscle, which is the largest tissue of salmonid fish. It makes up around 35% of the body mass [19] and so is presumed to be a tissue that accounts for a large proportion of the whole-organism energetic requirements. The fish were humanely culled using an overdose of benzocaine (1 g l^−1^ in 0.95% ethanol solution) and immediately weighed on an electronic balance (E2000D; Sartorius, Göttingen, Germany). Muscle tissue (570.4 ± 9.1 mg) was then excised within 2 min of death and transferred to 10 ml of ice-cold isolation buffer (100 mM sucrose, 50 mM Tris, 5 mM MgCl_2_, 5 mM EGTA, 100 mM KCl, 1 mM ATP, pH 7.4).

### (b) Mitochondrial isolation

Isolates of fish muscle mitochondria were prepared using a modified version of the protocol described by Dawson and Scott [20]. The muscle extracts were minced and then gently homogenized with six passes of a Teflon-on-glass homogenizer (100 r.p.m.). Homogenates were centrifuged at 1000 *g* for 10 min and filtered through cheesecloth. The filtrate was then centrifuged at 8700* g* for 10 min. These pellets were then resuspended in 10 ml of storage buffer (0.5 mM EGTA, 3 mM MgCl_2_, 60 mM potassium methanesulphonate, 20 mM taurine, 10 mM KH_2_PO_4_, 20 mM HEPES, 110 mM sucrose, 0.02 mM vitamin E succinate, 2 mM pyruvate, 2 mM malate, pH 7.1) and centrifuged again at 8700 *g*. The pellets were finally resuspended in 1000 µl of storage buffer. Part of this mitochondrial isolate was kept on ice until mitochondrial physiology was measured and the rest was stored at −80°C for later use in protein assays.

### (c) Mitochondrial physiology measurements

Mitochondrial physiology was measured at 7 °C using high-resolution respirometry and fluorometry in an Oxygraph−2k with O2k-Fluorescence module (Oroboros Instruments, Innsbruck, Austria). Isolates of fish muscle mitochondria were added to the respirometry chamber (using approx. 230 µg mitochondrial protein) that contained respiration buffer (MiR05; 0.5 mM EGTA, 3 mM MgCl_2_, 60 mM potassium lactobionate, 20 mM taurine, 10 mM KH_2_PO_4_, 20 mM HEPES, 110 mM sucrose, 1 g l^–1^ fatty acid-free BSA; pH 7.3). Respiration rate was measured as the rate of decline in O_2_ concentration in the chamber, which contained a final volume of 2 ml. ROS emission rates were measured simultaneously (i.e. in parallel with mitochondrial oxygen consumption rates) at each step of the titration process as described in Dawson *et al*. [11] by fluorescence detection of resorufin (excitation wavelength of 525 nm, AmR filter set, Oroboros Instruments), which is produced from hydrogen peroxide (H_2_O_2_) and Ampliflu Red (10 µM; Sigma-Aldrich, Oakville, Ontario, Canada) in a reaction catalysed by horseradish peroxidase (3 U ml^–1^) and superoxide dismutase (SOD; 22.5 U ml^–1)^. Calibration of the fluorescent resorufin signal was conducted twice during the experiment with the addition of exogenous H_2_O_2_.

Mitochondrial respiration and ROS emission in the absence of ADP were measured first after malate (2 mM) and pyruvate (5 mM; L_N_) injections. Then the ADP/O ratio was determined as described previously [21,22] by measuring the respiration induced by the addition of a limiting amount (125 μmol l^–1^) of ADP, with ADP/O calculated as the concentration of ADP injected in μmol divided by the concentration of O_2_ consumed (μ-atoms). Once the ADP was consumed and oxygen consumption rates stabilized, a saturating amount of ADP (5 mM) was then added to stimulate full oxidative phosphorylation (OXPHOS) via complex I in the presence of pyruvate and malate (P_PM_). Glutamate (10 mM) and then succinate (25 mM) were added next to determine the maximum OXPHOS capacity via complexes I + II (maximum OXPHOS, sometimes elsewhere referred to as P_PMGS_), with simultaneous measurement of ROS production. Exogenous cytochrome c (10 µM) was added to assess the integrity of the outer mitochondrial membrane; the effect on respiration was always modest (approx. 4% on average and always <10%), indicating that the method of isolating the mitochondria was robust. The addition of oligomycin was used to measure LEAK state respiration (sometimes elsewhere referred to as L_Omy_). Addition of antimycin A revealed non-mitochondrial or background oxygen consumption, which was subtracted from all other measurements. Mitochondrial respiration and ROS emission rates are expressed per mg mitochondrial protein, which was measured using the Bradford assay (Bio-Rad Laboratories, Watford, UK).

The respiratory control ratio was established by calculating the ratio of the respiration rate following the addition of pyruvate, malate, glutamate, succinate and ADP (P_PMGS_) relative to the leak respiration state (L_Omy_; addition of oligomycin). Net phosphorylation efficiency (P_efficiency) was calculated as described in Dawson *et al*. [11]:


P_efficiency=1–(1/RCR),orP_efficiency=1–(LEAK/OXPHOS)

This index of mitochondrial efficiency ranges between the hypothetical values of 0 (where the mitochondria would produce no ATP even when all substrates are available) and 1 (where there is no leak respiration, so that all of the mitochondrial oxygen consumption would be devoted to ATP production). The ratio of ROS emission to oxygen consumption during maximum OXPHOS respiration (P_PMGS_) was calculated as described in Du *et al*. [22]. The reagents used in this section, and elsewhere throughout, were obtained from Sigma-Aldrich (Gillingham, Dorset, UK) unless otherwise stated.

### (d) Statistical analysis

PIT tag data were extracted from raw data files containing the time, date and RFID number (see electronic supplementary material, 1, for more detailed information). Reads were taken every 16 seconds so each instance of the PIT tag being recorded in the shelter was therefore assigned a duration of 16 seconds. From this, the total time that fish spent in the shelter was determined as 16 seconds × the number of readings recorded by the RFID reader contained within the 3D-printed shelter. The proportion of time spent in the shelter was determined by calculating the total time that fish spent in the shelter as a fraction of the total duration of the experimental trial. Hourly sheltering time was determined by the number of readings between 1 second after each hour and the start of the next hour (e.g. 1 h + 1 second to 2 h). Daytime sheltering time was determined by the number of readings recorded between 8 h00 (+1 second) and 18 h00, whereas night-time sheltering time was determined by the number of readings recorded between 19 h00 (+1 second) and 8 h00.

We used generalized linear mixed models using template model builder (R v. 4.1.2; see http://www.R-project.org/; package glmmTMB) to determine the relationships between individual variation in mitochondrial physiological parameters (P_efficiency, ADP/O ratio, maximal OXPHOS respiration (P_PMGS_) rate and ROS release rate in muscle) and the proportion of time fish spent in the shelter over the week-long monitoring period. The model used a beta family to account for zero inflation and included the proportion of time in shelter (either by day, by night, or in total) as the dependent variable with P_efficiency, ADP/O ratio, maximal OXPHOS respiration, ROS release rates of muscle and body mass as covariate explanatory variables. The conditional effects for significant mitochondrial parameters and sheltering time were represented visually using ggpredict and ggplot (R v. 4.1.2; http://www.R-project.org/; packages ggeffects and ggplot2). The Pearson’s product–moment correlation between the ADP/O ratio and P_efficiency was calculated using cor.test; R v. 4.1.2; http://www.R-project.org/; package corrr). Processing batch was included as a random effect in all mixed models to control for the order in which fish were processed. Repeatability of an individual fish’s sheltering time between days was assessed for both day- and night-time (R v. 4.1.2; http://www.R-project.org/; package rptR) using 1000 bootstraps and a Poisson distribution. The differences in mean time spent sheltering between efficiency groups (top 25% versus bottom 25% of fish ranked for mitochondrial efficiency) were evaluated using a Student’s *t*‐test (R v. 4.1.2; http://www.R-project.org/; package stats). Significance level was set to *p *< 0.05 in all statistical tests. See electronic supplementary material, 1, for outcomes of model fitting, variance and dispersion parameters. All data generated in this experiment can be found in electronic supplementary material, 2.

## 3. Results

**Figure 1.**
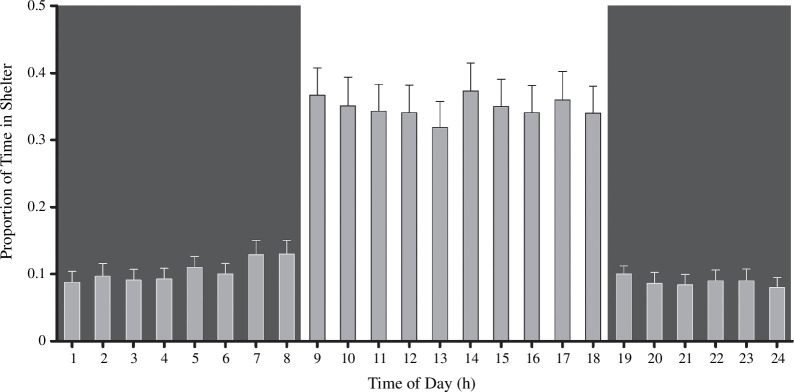
Proportion of time spent in the shelter for each hour during the day. Fish spent a greater portion of their time in the shelter during the day (white background) than at night (black background).

**Figure 2.**
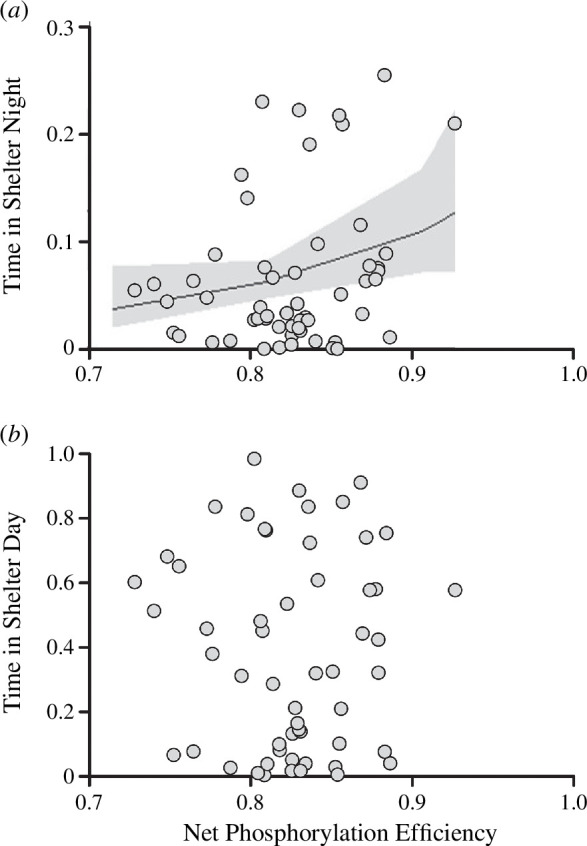
Relationship between the proportion of time salmon spent in the shelter during the (*a*) night and (*b*) day and mitochondrial net phosphorylation efficiency in the muscle of juvenile salmon acclimated to winter conditions (7°C). Black line shows the significant conditional effect based on the statistical analysis given in table 1, with 95% confidence intervals (grey area).

Fish spent a higher proportion of their time in the shelter during the day (mean = 39.6%; range = 0.61–98.4%; *n* = 55) than they did at night (mean = 10.40%; range = 0.09–38.60%; *n* = 55; figure 1). The time spent sheltering by each individual was highly repeatable across the full 7 days for both day (*R* = 0.81; s.e. = 0.036; confidence interval (CI) = 0.725–0.866; *p *< 0.001) and night (*R* = 0.75; s.e. = 0.045; CI = 0.644–0.819; *p *< 0.001). At night, shelter use was significantly related to P_efficiency, whereby juvenile salmon with more efficient mitochondria spent more time in the shelter (figure 2*a*, *p* = 0.045; table 1). This positive relationship between the relative amount of time spent sheltering and mitochondrial efficiency was not significant during the day (*p* = 0.843; *n* = 55; table 1), nor when the time spent sheltering by day and by night were combined (*p* = 0.385; *n* = 55; table 1). After taking P_efficiency into account, maximum OXPHOS respiratory capacity, ROS release rates and body mass showed no significant effects on the amount of time that fish spent sheltering by night, by day or in total (table 1). P_efficiency showed a significant, positive relationship with ADP/O ratio (electronic supplementary material, figure X; *t*_53_ = 3.062; *r* = 0.3878; 95% CIs = 0.1364–0.5921; *p* = 0.0034), suggesting that the two measures of efficiency are related.

**Table 1.** (*a,b*) Mixed-model analyses for the proportion of time spent in the shelter (by (*a*) night and (*b*) day) by juvenile salmon as a function of body mass and muscle mitochondrial properties (net phosphorylation efficiency (P_efficiency), maximum OXPHOS respiration rate (P_PMGS_) and ROS release rates during maximum OXPHOS respiration). (*c*,*d*) Mixed-model analyses for the total proportion of time spent in the shelter by juvenile salmon as a function of body mass, time period (day and night) and muscle mitochondrial properties (net phosphorylation efficiency (P_efficiency), maximum OXPHOS respiration rate (P_PMGS_) and ROS release rates during maximum OXPHOS) with (*c*) and without (*d*) the interaction of time period and P_efficiency. Processing batch was included in all models as a random effect to control for the order in which fish were processed. Bold denotes significant terms.

dependent variable	source of variation	parameter estimate ± s.e.	statistical results
(*a*) proportion of time in shelter (night)	intercept	−6.177 ± 2.932	
	body mass	−0.074 ± 0.092	*Z*_47_ = −0.796, *p* = 0.426
	**P_efficiency**	**6.937 ± 3.457**	***Z*_47_ = 2.007, *p* = 0.045**
	log(OXPHOS)	*−0.133 ± 0.320*	*Z*_*47*_ *= −0.414, p = 0.679*
	ROS release during OXPHOS	−0.681 ± 0.489	*Z*_47_ = −1.392, *p* = 0.164
	ADP/O	−0.324 ± 0.440	*Z*_99_ = −0.735, *p* = 0.462
(*b*) proportion of time in shelter (day)	intercept	−2.583 ± 0.522	
	body mass	−0.130 ± 0.115	*Z*_47_ = −1.138, *p* = 0.255
	P_efficiency	0.745 ± 3.764	*Z*_47_ = 0.198, *p* = 0.843
	log(OXPHOS)	0.197 ± 0.415	*Z*_47_ = 0.474, *p* = 0.636
	ROS release during OXPHOS	−0.129 ± 0.531	*Z*_47_ = −0.243, *p* = 0.808
	ADP/O	*−0.116 ± 0.522*	*Z*_*47*_ *= −0.223, p = 0.824*
(*c*) proportion of time in shelter (total)	intercept	−1.948 ± 3.047	
	body mass	−0.127 ± 0.095	*Z*_99_ = −1.344, *p* = 0.179
	P_efficiency	*1.054 ± 3.436*	*Z*_*99*_ *= 0.307, p = 0.759*
	log(OXPHOS)	0.048 ± 0.348	*Z*_99_ = 0.137, *p* = 0.891
	ROS release during OXPHOS	−0.289 ± 0.480	*Z*_99_ = −0.603, *p* = 0.547
	ADP/O	−0.120 ± 0.463	*Z*_99_ = −0.260, *p* = 0.795
	time period	−4.827 ± 2.799	*Z*_99_ = −1.724, *p* = 0.085
	time period × P_efficiency	4.851 ± 3.371	*Z*_99_ = 1.439, *p* = 0.150
(D) proportion of time in shelter (total)	intercept	−3.564 ± 2.806	
	body mass	−0.125 ± 0.093	*Z*_100_ = −1.339, *p* = 0.181
	P_efficiency	2.952 ± 3.144	*Z*_100_ = 0.939, *p* = 0.348
	log(OXPHOS)	0.047 ± 0.344	*Z*_100_ = 0.138, *p* = 0.890
	ROS release during OXPHOS	−0.261 ± 0.473	*Z*_100_ = −0.552, *p* = 0.581
	ADP/O	−0.104 ± 0.456	*Z*_100_ = −0.227, *p* = 0.820
	**time period**	***−0.799 ± 0.160***	***Z*_*100*_ *= −5.004, p = 5.62 x 10*^*–7*^**

**Figure 3.**
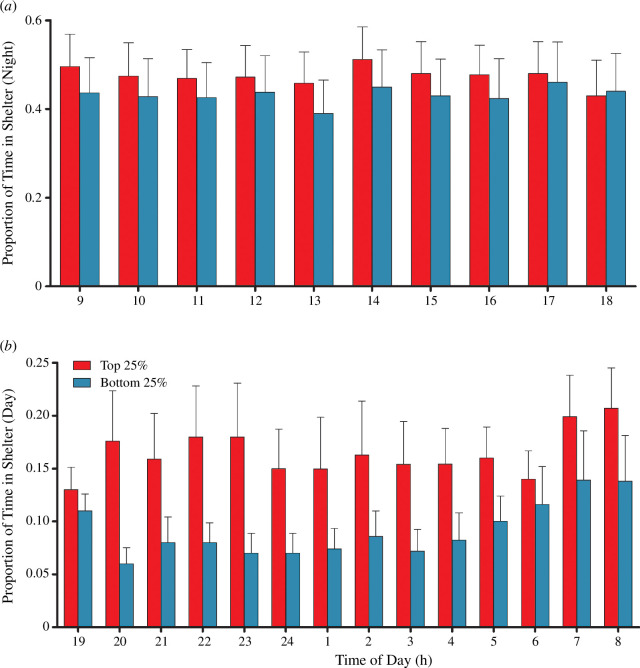
Proportion of time in shelter (+CI) for the most efficient (top 25%—red) and least efficient fish (bottom 25%—blue) for each hour during the (*a*) night and (*b*) day (significant main effect of efficiency using a two-factor ANOVA for night: *F*_1,13_ = 31.27; *p* < 0.0001; non-significant effect for day: *F*_1,9_ = 1.50; *p* = 0.2221).

To further explore the relationship between mitochondrial P_efficiency and sheltering time, we evaluated the behavioural differences between the fish with the most (top quartile) and least (bottom quartile) efficient mitochondria. Salmon with the most efficient mitochondria spent, on average, nearly twice the amount of time in the shelter at night in comparison to the least efficient fish (*p *< 0.0001; *n* = 14 for both the top 25% and bottom 25%; figure 3*a*; table 2). There were no significant differences in total or daytime sheltering between fish with the most and least efficient mitochondrial (*p* = 0.2221; top 25% *n* = 14; bottom 25% *n* = 14; figure 3*b*; table 2).

**Table 2.** Time spent in the shelter (hours) for salmon of either the top quartile (25%) or bottom quartile (25%) in terms of mitochondrial net phosphorylation efficiency (P_efficiency). * denotes a significant difference between the top and bottom quartile fish.

parameter	efficiency group	muscle	
		mean	*p*‐value
time in shelter (night)	top quartile	18.16 ± 3.44*	**0.0325**
	bottom quartile	**8.95 ± 2.10**	
time in shelter (day)	top quartile	31.22 ± 5.12	0.9006
	bottom quartile	30.25 ± 5.73	
time in shelter (total)	top quartile	49.38 ± 6.10	0.2670
	bottom quartile	39.20 ± 6.57	

## 4. Discussion

The results of this study show that juvenile salmon spend more time in the shelter during the day in comparison to the night. The time spent sheltering at night, but not during the day, showed a significant correlation with mitochondrial P_efficiency, where the most efficient individuals spent a greater amount of time in the shelter. However, individuals exhibited a wide range of variation in both mitochondrial efficiency and sheltering times during both the night and the day. The individuals with the highest P_efficiency also had similarly elevated ADP/O ratios and lower overall LEAK state respiration, but no difference in their maximum OXPHOS capacity or ROS release rates in comparison to less efficient individuals. This suggests that sheltering time and possibly foraging patterns could be dictated, in part, by how efficiently individual fish can produce ATP via their mitochondria.

### (a) Sheltering to survive

Fish showed a preference towards staying in the shelter during the day, spending on average a third of the allowable time in the shelter, whereas they spent only a tenth of the allowable time in the shelter at night. This is in line with previous findings, which found that juvenile salmonids in winter spend proportionally more time hiding in streambed shelters during the day and typically emerge at night to feed [13,23,24]. Individuals with the most efficient mitochondria spent relatively more time in the shelter during the night, but not during the day, in comparison to those individuals with less efficient mitochondria. It is possible that fish with more efficient mitochondria require less food to satisfy their energetic requirements, as found at other times of the year when they are growing actively [10,11]; this would thereby reduce their need to forage at night. This phenomenon may be more pronounced in the wild, since it has earlier been shown that fish with lower whole-organism oxygen consumption rates were able to spend more time in shelters and less time in exposed parts of a semi-natural stream [25]. In fact, the ability to obtain a shelter may be paramount to survival, as a study of juvenile Atlantic salmon showed that even at very high densities, shelters or refuges were typically only occupied by a single individual [26]. The competition for shelters is also seen within a given family of fish, where juvenile salmon did not share access to winter refuges with their siblings [27]. In addition, seasonal variation in temperature affects activity patterns, with a shift towards more foraging and less sheltering with an increase in temperature within a population [15]. However, it may be possible that population-level variation in sheltering is dictated by thermal adaptation, where fish populations from warmer streams are less likely to use shelters [28], possibly owing to a propensity for growth in the warmer months and a general increase in metabolic requirements at warmer temperatures. This would further benefit fish with more efficient mitochondria, since previous work on the closely related brown trout *Salmo trutta* discovered that an individual’s mitochondrial efficiency seemed to determine growth rate only at higher temperatures [11]. However, ectothermic predators may also have elevated metabolic needs in a warmer climate, so energetic demand and predation risk might both be elevated in warmer months.

Conversely, shelter availability may influence energetics independently of energy production efficiency. Finstad *et al*. [29] demonstrated that juvenile salmon with greater access to shelters lost less mass over winter, with the effect being strongest in larger fish. It has been calculated that the greater preference for sheltering during the day and foraging at night arises from a more favourable ratio of predation risk to feeding rate, owing to the much greater predation risk by day outweighing the slightly greater daytime feeding efficiency [13]. Fish should therefore feed primarily at night and only for as much of the day as is necessary to achieve the required food intake. Food requirements are greater at warmer temperatures, and so the fish should increase their daytime activity as temperature increases [15] since the benefits of risky feeding increase as nutritional requirements rise [2,3]. The observed relationship between sheltering time and mitochondrial efficiency may therefore be more pronounced in the face of global warming, where more efficient fish may be able to attain a larger size more quickly and remain sheltering for longer, ultimately reducing predation risk and improving their chances of survival.

### (b) Variation in mitochondrial efficiency depends on mitochondrial ATP production

The positive relationship between sheltering time and mitochondrial efficiency demonstrates that cellular energy production and behaviour may be linked through mitochondrial ATP production efficiency. However, we set out to ascertain what the underlying mechanisms were to account for this individual variation in mitochondrial efficiency. We observed a positive correlation between mitochondrial ADP consumption efficiency (ADP/O ratio; a good proxy of mitochondrial ATP production) and P_efficiency in the muscle of juvenile salmon. This relationship has not been widely studied in the past; however, the positive relationship between the ADP/O ratio and P_efficiency shows that ATP production efficiency is likely linked to oxygen consumption efficiency. Mitochondria are thought to be responsible for consuming almost 90% of the oxygen used by the whole organism [30]. Therefore, it is possible that measuring the fate of oxygen consumed at the level of the mitochondria (i.e. LEAK vs. OXPHOS) might provide the most accurate measure of efficiency. A change in LEAK state respiration has previously been shown to relate to variations in metabolic rates, where fish with greater mitochondrial LEAK have higher overall metabolic rates [9]. In this same study by Salin *et al*. [9], it was suggested that decreased mitochondrial leakiness would have a trade-off with higher ROS release rates. Our results do not show this pattern. However, ROS release rates are decidedly lower in cold-acclimated salmonids [11,31], which may suggest that this trade-off in increased ROS may only be observable at high temperatures.

## 5. Conclusions

In conclusion, our study has demonstrated that the time spent sheltering at night, but not during the day, is predicted by mitochondrial phosphorylation efficiency, where the most efficient individuals spent a greater amount of time in the shelter. Future work should focus on how such individuals can maintain efficient mitochondrial function while minimizing ROS release. However, it is possible that lower temperatures, as encountered in winter, are minimizing mitochondrial ROS release rates [11,31]. Regardless, the findings of this study suggest that individual heterogeneity in cellular function may drive variation in the foraging or sheltering patterns of aquatic animals. Future work aimed at appreciating these relationships will help gain further insight into the contribution of variation in mitochondrial physiology to aerobic performance and behavioural adaptations, which has implications for selection pressures acting on wild populations.

## Data Availability

All data used in the study can be found in the electronic supplementary material [32].
